# Assessment of left ventricular torsion in long axis view in patients with chronic stenosis of coronary arteries

**DOI:** 10.22088/cjim.11.1.21

**Published:** 2020

**Authors:** Ali Jabari, Manijeh Mokhtari-Dizaji, Zahra Arab- Bafrani, Elham Mosavi, Leili Mahani, Mohammad Mostakhdem Hashemi

**Affiliations:** 1 Ischemic Disorders Research Center, Golestan University of Medical Sciences, Gorgan, Iran; 2 Department of Anesthesiology and Critical Care Medicine, Golestan University of Medical Sciences, Gorgan, Iran; 3 Department of Medical Physics, Tarbiat Modares University, Tehran, Iran; 4 Department of Medical Physics, Faculty of Medicine, Golestan University of Medical Sciences, Gorgan, Iran.; 5 Metabolic Disorders Research Center, Golestan University of Medical Sciences, Gorgan, Iran; 6 Department of Medical Microbiology, Kerman University of Medical Sciences, Kerman, Iran; 7 Department of Radiation Oncology, Seyed Al-Shohada Hospital, Isfahan University of Medical Sciences, Isfahan, Iran

**Keywords:** Coronary artery disease, Echocardiography, Left ventricular torsion, Speckle tracking, Block matching algorithm.

## Abstract

**Background:**

Left ventricular torsion is one of the most important biomechanical parameters of heart that routinely is measured in short axis view. A review of the literature has indicated that assessment of left ventricular torsion in short axis view has some limitations. In the present study, we evaluated whether torsion angle assessment in long axis view can be used as a diagnostic biomechanical marker in patients with coronary artery disease (CAD).

**Methods:**

We assessed 20 males and 15 females who suffered from CAD and 24 healthy males and females. Two dimensional echocardiography images were scanned in cine loop format position throughout four cardiac cycles at basal and apical levels in the long axis view (4CH). Peak torsion angle in long axis view was obtained by speckle tracking method under block matching algorithm.

**Results:**

In long axis view, peak torsion angle and time of peak torsion angle were similar in female (34.87±5.8˚, 287±18ms) and males (33.26±5.60˚, 295 22ms) while they were significantly decreased and increased in CAD patients (female: 24.91±3.5˚, 345±26ms and male: 24.15±2.16˚, 358±24 ms) in comparison to healthy subjects. The peak torsion angle reduction in CAD patients was a subsequent of reduced rotation angle of basal and apical levels (P<0.001).

**Conclusion:**

The results showed that sex difference did not influence torsion angle of the left ventricle. It is concluded that left ventricular torsion assessment in long axis view has the potential to distinct patients with CAD from healthy subjects in routine echocardiography evaluation.

Coronary artery disease (CAD) is the most common type of heart disease and mortality causes in the world. This disorder develops with stiffness and narrowing the coronary arteries (1, 2). Angiography is a standard method for the evaluation of coronary artery disease but its constant usage is not appropriate due to cumbersome, time consuming and invasiveness. With respect to this fact, nowadays estimation of myocardial mechanical parameters is desired for diagnosis of cardiovascular function abnormality. Based on the recent studies, coronary stenosis has changed mechanical parameters of myocardium (3, 4). Recently, torsion angle of left ventricular (LV) that is dependent to myocardial fiber orientation, structure, and function is proposed as one of the essential LV mechanical parameters which can show early changes in LV function[6]. Clock wise rotation of the base and counter-clock wise rotation of the apex of the heart around its long axis, cause the heart torsion during systolic phase (5, 6).

Speckle tracking echocardiography (STE) is a new method for the assessment of myocardial motion, independent of the angle between tissue and ultrasound beam (7, 8). Notomi et al (9) used STE under block matching algorithm for assessment of left ventricular torsion in short axis view. Recent studies have shown the alternation of LV torsion in short axis view of patient with diastolic dysfunction (10), Dilated cardiomyopathy (DCM) (9), and CAD (11). 

There is a controversy on the torsion angle magnitude among different studies due to the dependency of basal and apical rotation on the place of reference levels in short axis view and assuming the circularity of LV (12,13). Moreover, though-plane displacement and lower acoustic conditions in short axis view rather than long axis view cause misestimation of LV torsion magnitude by speckle tracking (9, 13-15). 

Recent studies have shown that the assessment of myocardial movement along the long-axis view of the LV has some advantages in comparison to short axis view and covers its limitation (16, 17). In our previous study, we demonstrated that rotation angles in the long axis view have almost similar behavior in comparison to short-axis view (17). 

Regarding to the relation of left ventricular mechanical dyssynchrony specially torsion angle with regional fibrous deformation that may occur in CAD patients and advantages of torsion angle assessment from longitudinal rather than short axis view, the aim of present study was to assess whether LV torsion parameters in long axis view can be proposed as diagnostic biomechanical marker of patients with CAD. Also, due to the hypothesis of torsion angle dependency to LV remodeling which itself is affected by gender (18), we evaluated this issue for both the female and male subjects.

## Methods

**Study population**: We assessed 20 males and 15 females with diagnosed CAD and 24 healthy subjects (14 males and 10 females). The patients with atypical chest pain, electrocardiography changes, and positive exercise test were enrolled in the study besides they have documented ischemic heart disease (IHD) based on cardiac catheterization study. They did not have any kind of valvular or congenital heart disease. Healthy subjects did not have any history of heart disease such as myocardial infarction, ventricular arrhythmia, heart failure hypertension, diabetes, drug consumption, and any kind of coexisting disease. They had a normal physical examination, electrocardiogram (ECG), echocardiography and did not undergo coronary angiography selected based on Framingham study (19, 20). 

This study was performed in the heart center of Amiralmomenin Hospital-Kordkuy (from August to November 2018) through random sampling and informed consent was taken from all subjects prior to participation in the study.

**Instrument**: 2-1- A vivid GE echocardiography system (Horten, Norway) with M3S transthoracic sector transducer (1.5-4MHz) was used for recording the original images. 2-2- Post processing: 4 cine-loops from each standard views were transferred to personal computer (PC) computer for post processing by experimental software, Block matching algorithm under sum absolute difference (SAD) SAD criterion, that programmed in MATLAB software Version 7.0.1 (Math Software Co., Mathworks, USA) (11, 17, 21).

**Echocardiography Recordings: **For each subject, we scanned long axis views using high frame rate (60-70 frame per second) harmonic B-mode imaging with transmitted and received frequency 1.7 MHz and 3.4 MHz, respectively. In long axis view, basal and apical levels have been in 1/2 high and 1/3 low of left ventricular axis, respectively (12). The subjects were imaged at rest and lying in the lateral decubitus position. 

Two-dimensional ECG was superimposed on the images and end-diastole was considered at the peak R-wave of the ECG. The LV end-diastolic/systolic diameters (d_LVED_/d_LVES_), interventricular septal diastole thickness (T_IVSD_), posterior wall thickness (T_PW_) were measured in the long-axis view of LV, also LV ejection fraction (LV-EF) were calculated using Simpson's biplane method by measuring end-diastolic and end-systolic volumes in 2D images. 2D Echocardiography imaging was performed using standard transthoracic apical two and four-chamber views according to guidelines of the American Society of Echocardiography (ASE) (22).

**Figure 1:**
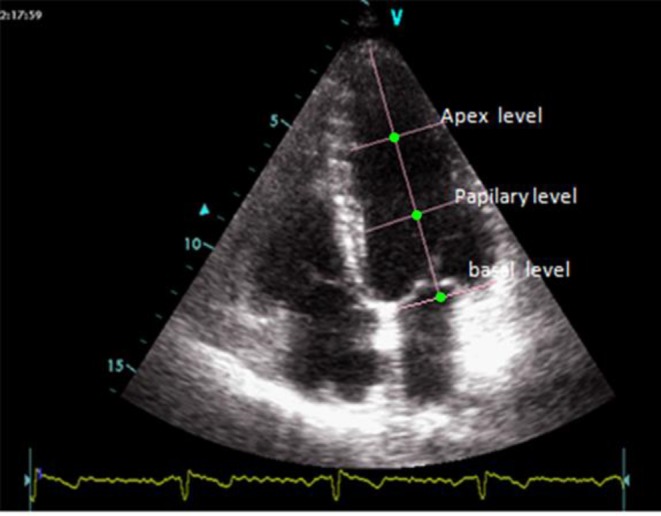
Central of LV (green dot) in long axis view at different levels

**Image analyzing**: Measuring LV torsion in long axis view by STE under block matching algorithm was performed as reported in our previous study (17). Briefly, after selecting the best quality two dimensional images and converting them to consecutive frames, displacement-time curve of septal myocardial walls in basal and apical levels were drawn using block matching algorithm (11, 17, 23). The frame of end diastolic phase was determined according to recorded ECG and displacement-time curve. After that, The center of left ventricular in long axis views was determined by drawing diameter of apical and basal levels in end diastolic phase frame (fig. 1) (17). 

Basal/ apical- LV rotation in long axis view at septal wall were calculated based on the following equation, Eq. 1. 

Eventually torsion angle was defined as the net difference between the apical and basal LV rotation Eq. 2.

${\varphi (t)}_{\mathrm{long}-\mathrm{axis}}=(A\tan\left(\frac{Z_{t}-Z_{\mathrm{center}}}{x_{t}-x_{\mathrm{center}}}\right)-A\tan\left(\frac{Z_{t_{o}}-Z_{\mathrm{center}}}{x_{t_{o}}-x_{\mathrm{center}}}\right)))$

$x_{t}$ and $Z_{t}$ are t-time displacements in X and Z directions. $x_{t_{o}}$ and $Z_{t_{o}}$ are end diastolic displacements in X and Z directions and so $x_{\mathrm{center}}$ and $Z_{\mathrm{center}}$ are the center of the LV in long axis view.

${\emptyset (t)}_{\mathrm{long}-\mathrm{axis}}={\emptyset (t)}_{\mathrm{apex}-\mathrm{axis}}-{\emptyset (t)}_{\mathrm{base}-\mathrm{level}}$

 Eq. 2

${\emptyset (t)}_{\mathrm{apex}-\mathrm{level}}$ and ${\emptyset (t)}_{\mathrm{base}-\mathrm{level}}$ are rotation angles of apical and basal levels in long axis view, respectively.

Finally, we measured the magnitude of peak torsion and calculated the time to peak torsion. 

**Statistical analysis: **We employed statistical analysis software SPSS 16.0 in our study. Data were expressed as mean±SD). Data were tested for normal distribution and homogeneity of variance by the Kolmogorov-Smirnov test (K-S). The difference between two groups was tested by unpaired t-test and Mann Whitney U-tests in case of parametric and non-parametric data with confidence level of 95%.

**Ethics: **This study was approved by the Regional Committee of Ethics in Research of Golestan University of Medical Sciences with grant number: IR.GOUMS.REC.1397.278.

## Results

**Conventional ultrasound examination of healthy subjects and CAD patient:** The clinical characteristics and conventional echocardiographic values of the subjects were summarized in table. 1. There were no significant differences between age, sex, heart rate and body mass index (BMI) of healthy persons and CAD patients (p>0.05). Also, there were no significant differences between posterior wall, interventional septal thickness, end systolic diameter, and ejection-fraction of two mentioned groups (p>0.05).

**Myocardial Motion Analysis:** The horizontal and vertical myocardial septal movements in basal and apical levels in long axis views of healthy subjects and CAD patients are depicted in table 2. Independent- samples t-test showed there were no significant differences between septum wall movements of healthy subjects and CAD patients in all directions except horizontal displacement of apical view. 

**LV Rotation and Torsion angle:** Rotation and torsion angles in long axis views were calculated through extraction of the instantaneous changes of the vertical and horizontal displacement vector throughout a cardiac cycle using block matching algorithm and determination of the desired level center in end diastolic phase frame. As seen from the apical level, two groups had the same rotational behavior, as in systolic phase rotated clock wise in basal level and counter-clock wise in apical level. 

**Figure 2:**
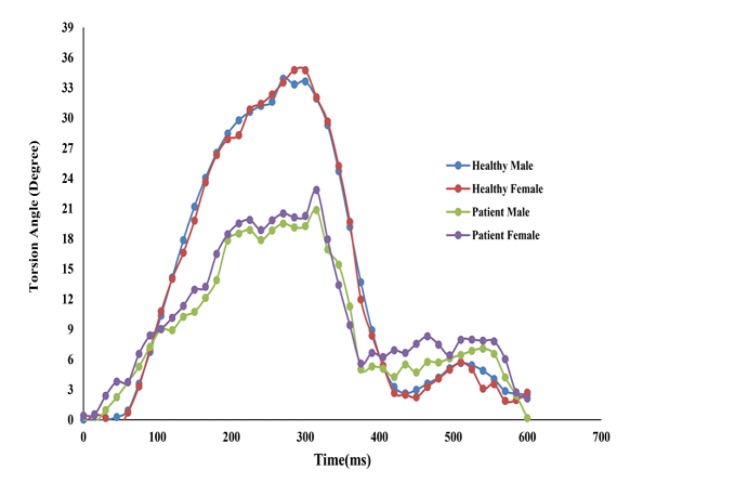
Torsion angle in healthy subjects and CAD patients during a cardiac cycle in long axis view

Torsion angle of the healthy subjects and CAD patients in long-axis views throughout a cardiac cycle for both males and females were demonstrated in fig. 2. As shown in table. 3, torsion angle peak of CAD patients in long axis view (female: 24.91±3.5, male: 24.15±2.16) were significantly reduced compared with healthy subjects (female: 34.87±5.8, male: 33.26±5.60), as the result of decreased apical and basal rotation angles.

**Time to torsion angle peak** **: **Torsion angle peak time in long axis view was significantly longer in CAD patients than healthy subjects, whereas there was no significant difference between heart rate of two mentioned groups (table 4). 

**Table 1:** Clinical characteristics and conventional 2-dimentional echocardiography in healthy subjects and CAD patients (mean±SD)

**Clinical Characteristics**	**Variables**	**Male **			**Female **		
		**Healthy subjects**	**CAD patient**	**P-value**	**Healthy subjects**	**CAD patient**	**P-value**
	Age (year)	47± 5	46±8	0.90	45±9	46±5	0.7
	BMI: Body mass index (kg/m^2^)	25±2	26±1	0.73	26±3	25±6	0.5
	Heart Rate (beats/min)	72±8	70±13	0.80	73±9	72±10	0.1
	Radial diastolic blood pressure (mmHg)	80±10	78±7	0.04	80±8	77±10	0.03
	Radial systolic blood pressure (mmHg)	113±10	135±9	0.00	109±12	136±8	0.00
**Conventional 2-dimentional Echocardiography**	LV End systolic diameter (mm)	31.42±4.2	32.68 ±4.3	0.83	29.5±5	26.2±8	0.04
	LV End diastolic diameter (mm)	49.80±3.90	45.6±3.7	0.02	43 ±2.22	38±3.22	0.04
	Interventricular septal diastole thickness (mm)	11.92±0.82	10.5±0.7	0.33	8.8±1.5	10.6±1.7	0.03
	Posterior wall thickness (mm)	11.10±1.11	10±0.000	0.94	8.6±1.8	11.6±1.9	0.04
	Stroke volume (ml)	59.97±13.98	44±14.9	0.00	53 ± 9.22	39±10.44	0.00
	Left ventricular ejection fraction percent (LVEF%)	58.31±2.48	52±2	0.06	62.2±2.33	57.33± 3.22	0.07

**Table 2:** Myocardial septal displacement at apical and basal level in healthy subjects and CAD patients at long axis view (X̅ ±SD)

**Variable**		**Male**			**Female**		
		**Healthy subjects**	**CAD patients**	**P-value**	**Healthy subjects**	**CAD patients**	**P-value**
Apical displacement	Horizontal	8.11± 2.24	5.75±2.23	0.026	9.33± 1.33	6.01±2.2	0.015
	Vertical	3.53±0.76	3.70±1.60	0.505	3.6± 1.2	3.54±08	0.42
Basal displacement	Horizontal	3.59 ± 1.54	3.67±1.76	0.691	3.76±1.48	3.66+0.45	0.52
	Vertical	10.44±2.12	9.07±2.23	0.140	11.2±3.14	10±1.5	0.08

**Table 3:** Rotation and torsion angle of healthy subjects and CAD patients in long axis view (X̅ ±SD):

**Variable**	**Male**			**Female**		
	**Healthy persons**	**Patient**	**P-value**	**Healthy subjects**	**Patient**	**P-value**
Basal rotation	18.51±3.41	13.24±1.31	0.000	19.4 ± 2.55	14.45±1.4	0.001
Apical rotation	14.74±2.91	10.91±1.91	0.000	15.33± 3.2	10.46±2.1	0.000
Torsion angle	33.26±5.60	24.15±2.16	0.000	34.87± 5.8	24.91±3.5	0.000

**Table 4:** torsion angle peak time in Healthy case and patient group (X̅ ±SD)

		**Healthy subjects**	**CAD patients**	**P-value**
Torsion angle peak time(ms)	Male	295±22	358±24	0.000
	Female	287± 18	345± 26	0.000

## Discussion

 Angiographic and CT angiographic imaging have been considered as the most important procedures for diagnosis of cardiovascular issues. However, their invasive nature and risk of ionizing radiation have restricted their repeated utilization. Nowadays, appropriate biomechanical parameters of myocardium based on modern echocardiography are proposed for many cardiac lesions. Based on recent studies, biomechanical abnormalities are seen in heart wall segments of patients with CAD (24, 25). One of the most important biomechanical parameters of heart is LV torsion. The heart torsion can influence the uniform distribution of stress along the myocardium wall so that the loss of torsion results in higher stress and strain on fibers and finally the function of heart is decreased along with the increased oxygen consumption. Diagnosis of early myocardial injuries is one of the potential features of heart torsion that is influenced by preload, afterload and contractility. Recent studies have exhibited that conditions such as ischemia decreases myocardium contraction and heart torsion (26, 27).

Wei et al. (28) indicated the LV peak torsion estimated by speckle tracking method was significantly reduced in patients with anterior myocardial infarction (AMI) compared with normal persons due to increased subendocardial stiffness in AMI patients. Moreover, researchers believe that in early stages of ischemia, myocardium, function abnormality is primarily detected in long axis view of the ventricle, (23). Therefore, in the present study, we estimated torsion angle in long axis view of CAD patients in comparison with healthy subjects. Prior to this assessment, we evaluated torsion angle-dependency to gender in long axis view. Contractility, independent of changes in ventricular volume, influences heart torsion by altering the contraction forces (26). As previously reported (29), higher LV-EF and greater systolic elasticity in women than men results in higher myocardial contractility. On the other hand, while males had greater LV volume than females, there were no sex differences in all metrically scaled volumes. In accordance with recent studies (30–32), there have been no sex differences in LV basal and apical rotation angles as well as torsion angle in long axis view.

Myocardial movements are able to affect the torsion angle of the heart; so large differences take place in the angle of torsion in response to even slight changes in the extent of myocardial movements. According to this fact, recent studies have proposed the recruitment of the maximum ventricular rotation angle in short axis view compared to the variations in movement as a predictive tool for potential heart muscle damages (9, 11). 

Comparisons between patient and healthy subjects showed the declined apical axial displacement in CAD patients in long axis views, while there was no significant reduction in basal level. It seems that this distinction is due to the nourishment of septal wall by multiple vessels in basal level and effects of stenosis in one vessel are diminished by other branches of coronary arteries. 

In the present study, we have demonstrated that LV rotation has been declined in basal and apical levels in long axis view under the condition of CAD occlusion. Such situation produces necrosis of the apex and anterior septum and consequently causes flatting of apical fiber and declining of apical rotation angle. Basal level may be inﬂuenced by the motion of adjacent muscle, and therefore neighboring myocardial regions have caused decline in basal rotation angle.

In conclusion present study indicated that LV torsion in long axis view decreased in CAD patient independent of gender. Therefore, it seems the assessment of LV torsion in long axis view has potential to distinct CAD patients form healthy subjects and might make the assessment more valuable in clinical and research cardiology. It is notable that our study investigated only the changes of LV myocardial septal wall rotation and torsion in long axis view. If imaging could be scanned with better spatial resolution, it would be possible to study other segments of myocardial wall. 
